# The effects of gait training using powered lower limb exoskeleton robot on individuals with complete spinal cord injury

**DOI:** 10.1186/s12984-018-0355-1

**Published:** 2018-03-05

**Authors:** Cheng-Hua Wu, Hui-Fen Mao, Jwu-Sheng Hu, Ting-Yun Wang, Yi-Jeng Tsai, Wei-Li Hsu

**Affiliations:** 1 Institute of Electrical Control Engineering, National Chiao Tung University, Hsinchu, Taiwan; 2 FREE Bionics, Hsinchu, Taiwan; 3 School of Occupational Therapy, College of Medicine, National Taiwan University, Taipei, Taiwan; 4 School and Graduate Institute of Physical Therapy, College of Medicine, National Taiwan University, Taipei, Taiwan; 5 Physical Therapy Center, National Taiwan University Hospital, Taipei, Taiwan

**Keywords:** Complete spinal injury, Exoskeleton robot, Gait, Training, Assistive device

## Abstract

**Background:**

Powered exoskeleton can improve the mobility for people with movement deficits by providing mechanical support and facilitate the gait training. This pilot study evaluated the effect of gait training using a newly developed powered lower limb exoskeleton robot for individuals with complete spinal cord injury (SCI).

**Methods:**

Two participants with a complete SCI were recruited for this clinical study. The powered exoskeleton gait training was 8 weeks, 1 h per session, and 2 sessions per week. The evaluation was performed before and after the training for (1) the time taken by the user to don and doff the powered exoskeleton independently, (2) the level of exertion perceived by participants while using the powered exoskeleton, and (3) the mobility performance included the timed up-and-go test, 10-m walk test, and 6-min walk test with the powered exoskeleton. The safety of the powered exoskeleton was evaluated on the basis of injury reports and the incidence of falls or imbalance while using the device.

**Results:**

The results indicated that the participants were donning and doffing the powered lower limb exoskeleton robot independently with a lower level of exertion and walked faster and farther without any injury or fall incidence when using the powered exoskeleton than when using a knee–ankle–foot orthosis. Bone mineral densities was also increased after the gait training. No adverse effects, such as skin abrasions, or discomfort were reported while using the powered exoskeleton.

**Conclusions:**

The findings demonstrated that individuals with complete SCI used the powered lower limb exoskeleton robot independently without any assistance after 8 weeks of powered exoskeleton gait training.

**Trial registration:**

Trial registration: National Taiwan University Hospital.

Trial registration number: 201210051RIB.

Name of registry: Hui-Fen Mao.

URL of registry: Not available.

Date of registration: December 12th, 2012.

Date of enrolment of the first participant to the trial: January 3rd, 2013.

**Electronic supplementary material:**

The online version of this article (10.1186/s12984-018-0355-1) contains supplementary material, which is available to authorized users.

## Background

For patients with spinal cord injury (SCI), the loss of mobility has a major effect on their daily life. Therefore, regaining functional mobility is highly desirable for people with SCI and is considered the top priority in rehabilitation treatments. Individuals with middle- or low-level thoracic SCI who are classified as ASIA Impairment Scale (AIS) A can perform functional activities such as sit-to-stand and walking while wearing a knee–ankle–foot orthosis (KAFO) and using assistive devices such as walkers or forearm crutches. However, walking with a KAFO is impractical in daily life because of the high energy expenditure and high level of skill required. For instance, the energy cost associated with bilateral-KAFO ambulation is 43% and 38% higher than that associated with wheelchair locomotion and normal walking, respectively [1]. In addition, high energy costs and inefficient walking (e.g., low walking speed) have been proposed to be the main factors for low compliance with the use of the ambulatory orthoses such as KAFOs [2]. Therefore, the goal of the assistive device is to reduce the level of exertion perceived by the users [3, 4].

When wearing a KAFO and using forearm crutches, individuals with paraplegia must rely only on his or her upper extremities to provide the extending moments required for lifting the upper body up and off the chair during sit-to-stand. Similarly, to walk with aids such as forearm crutches, individuals with paraplegia must rely heavily on their upper extremities to support their body weight while simultaneously ensuring smooth weight transfer to maintain their balance. Such movement patterns are not only inefficient, but they also potentially impose high joint loads on the shoulder and wrist [5], which can lead to subsequent upper extremity pathologies [6]. The required skill level with multiple muscles [7, 8] and joints and safety issues associated with performing such activities with compensatory movement patterns are also notable concerns.

Wearable powered exoskeleton robots have been developed to provide functional mobility to paraplegic patients [9]. Such an exoskeleton has several advantages over traditional KAFO or passive exoskeleton [10, 11]. For instance, motors at the hip and knee joints provide the hip and knee extending moments required for common functional activities such as sit-to-stand and walking. Therefore, patients can perform sit-to-stand and walking using a near-normal movement pattern rather than a compensatory movement pattern, which causes potential injury to their body. Hence, a lower skill level and lower energy are required to perform those functional activities with the assistance of the exoskeleton.

Commercialized exoskeletons have been developed for paraplegia such as the HAL [12], ReWalk [13], and EKSO [14]. HAL is working with active leg motion and they use an electromyography (EMG) measure to trigger the motion. However, the use of EMG is disputable. The EMG signal can be hard to interpret because of abnormal muscles activities; therefore the positioning is delicate for measurement. The link between the motion and the EMG is hard to define and not suitable for the population of complete SCI with statistics. ReWalk and EKSO are specially designed for SCI. However, the ankle joints of exoskeleton are completely passive, the balance stability is achieved by the user through the use of crutches and the leg motion. Moreover, the trigger button is set on a watch, and it is difficult to push the button and control crutches at the same time. Other muscle-driven exoskeleton was also developed which combined functional neuromuscular stimulation to activate the paralyzed muscles and generate joint torques for limb movements [15].

Although exoskeletons have been gradually introduced for both therapeutic training and personal use, the training effect and a standard training protocol are yet to be established. An exoskeleton must be safe, easy to use, and provide functional mobility to users without imposing high demands on them. For instance, the lower limbs of patients are forced to fit the shape of the exoskeleton to ensure a strong combination. A buckling force may be generated at the lower limbs because of the interactive effect between the restrict force and ground reaction force. To transfer the body from a wheelchair to an exoskeleton is difficult for and individual with complete SCI, because he or she must squeeze his or her body into the exoskeleton when donning the exoskeleton.

Therefore, to improve all aforementioned issues, the purpose of this study was to evaluate the feasibility and safety a newly developed lower limb powered exoskeleton robot by comparing it with a traditional KAFO when performing functional activities in individuals with a complete SCI, after an 8 weeks of gait training.

## Methods

### Lower limb powered exoskeleton robot

**Fig. 1:**
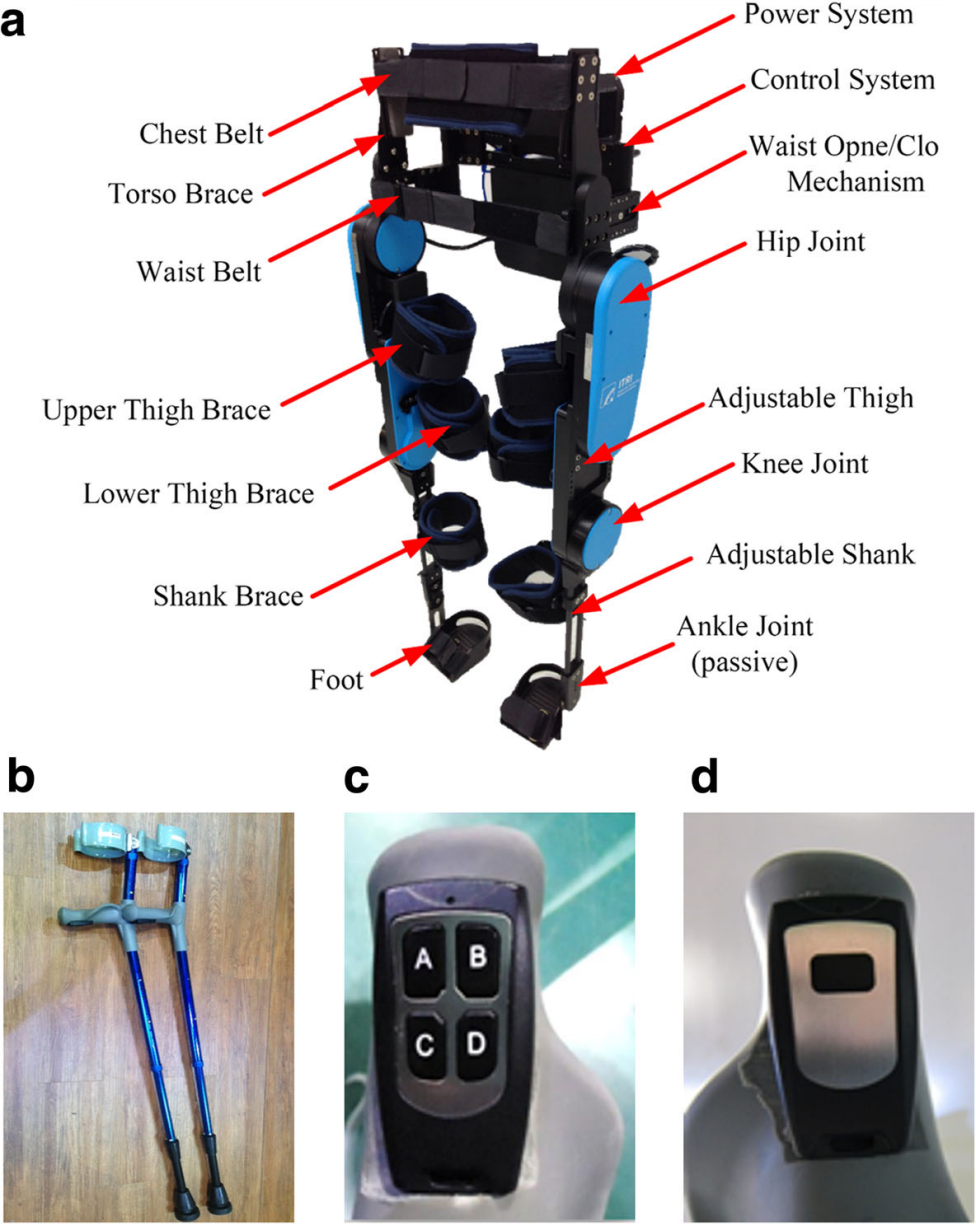
The powered exoskeleton robot (**a**), the forearm crutches (**b**), the right controller (**c**), and the left controller (**d**) that were used in the study

The powered exoskeleton was designed to provide full kinetic energy to assist paraplegic SCI patients with walking. Fig. 1 depicts the structure of the powered exoskeleton. According to Kim et al., the knee joint should provide a normalized peak torque of approximately 0.75 Nm/kg to accomplish sit-to-stand with arm support [16]. Crowell III et al. stated that the hip joint should provide a normalized peak torque of approximately 1.1 Nm/kg when walking at a normal speed. Moreover, the hip joint should allow for a sufficient walking speed of up to 1.0 m/s [17]. According to these criteria, a set commercial flat motors and speed reducers were used to construct the actuator for the hip and knee joints. The actuator can generate a rated torque of 35 Nm and a peak torque of more than 100 Nm for a 90-kg patient while walking and performing a stand-to-sit movement. The powered exoskeleton weighs only 20 kg (including the battery system) and allows for a maximum walking speed of 1.0 m/s.

To improve the usability, there are 3 special design criteria of the powered exoskeleton for users with SCI: convenient, safe, and burden-free use. These 3 criteria are described in details as follows:

#### Convenient to use

**Fig. 2:**
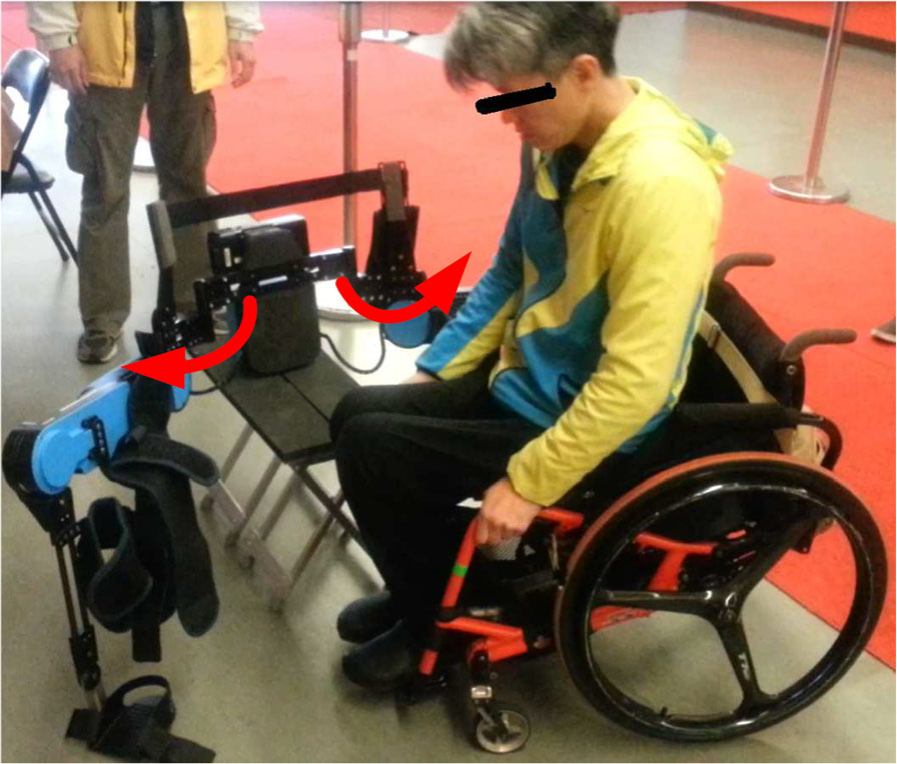
Waist open/close design of the powered exoskeleton

As shown in Fig. 2, the legs of the powered exoskeleton can rotate at the waist part. For individuals with SCI, this is a convenient feature for transferring between the powered exoskeleton and a wheelchair; and also for donning and doffing the powered exoskeleton independently. In addition, individuals with SCI can initiate the movement themselves by using a remote controller embedded in the handle part of the forearm crutches, instead of using a wristband. There are three motion modes for the powered exoskeleton: sit-to-stand, walking, and stand-to-sit. Sit-to-stand motion mode is the powered exoskeleton helps individuals with SCI to stand up from sitting posture. Walking motion mode is the power exoskeleton helps individuals with SCI to walk continuously or set steps with a predefined gait. Stand-to-sit motion mode is the power exoskeleton helps individuals with SCI to sit down from standing posture. To perform the three motion modes, the forearm crutches are required for supporting and balancing. Individuals with SCI can select the motion mode they intend to perform from the relative button of the remote controller embedded in the right-hand forearm crutch as shown in Fig. 1c and press the confirm button on the remote controller embedded in the left-hand forearm crutch as shown in Fig. 1d to execute the motion. As shown in Fig. 1c, button A is to sit-to-stand, button B is walking, and button C is stand-to-sit, respectively. For example, individuals with SCI presses button B on the right-hand forearm crutch to set the motion mode as walking, and the power exoskeleton will perform the walking motion after individuals with SCI presses the confirm button on the left-hand forearm crutch. Then individuals with SCI can stop walking by pressing the confirm button on the left-hand forearm crutch. Further, individuals with SCI can use the remote controller to lift the shanks at sitting posture for easily securing strap on the foot plate. For example, individuals with SCI can press the button C as shown in Fig. 1c to set the motion command as “lift the right shank up”, and then press the confirm button to lift the right shank up. After securing the strap, individuals with SCI can press the button C twice to set the motion command as “put the right shank down”, and then press the confirm button to put the right shank down.

#### Safe to use

**Fig. 3:**
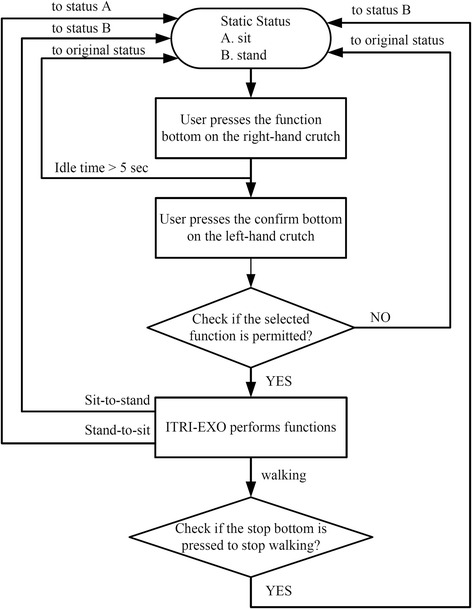
Flow chart of the powered exoskeleton control algorism

Figure 3 presents the mistake-proofing control flowchart so that the users can use the powered exoskeleton safely. Further, two safety functions are used to ensure the next step is safe to execute. One is to check the tilt angle and the angular velocity of the trunk of the user to ensure the center of mass is transferred well. The other is to check if the current of the motors is larger than the threshold when the swing leg collides with ground or the spasticity is too large to resist the motion. Moreover, the supporting part of the brace at each leg can be adjusted according to the shape of the user’s limbs. Therefore, the user’s limbs were not forced to fit the exoskeleton robot. This design prevented skin abrasions or pressure sore when using the exoskeleton robot to walk.

#### Burden-free to use

**Fig. 4:**
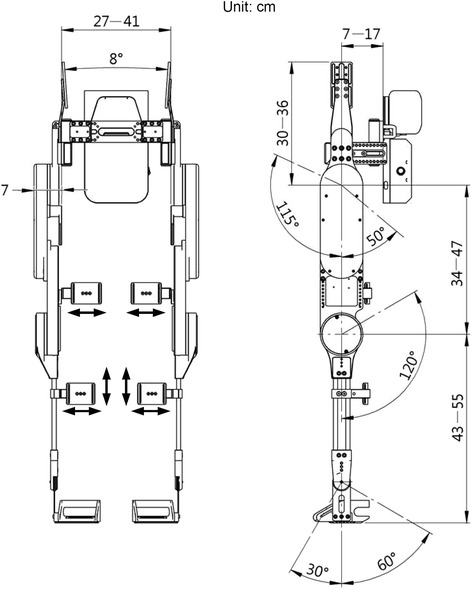
Aadjustable dimensions of the powered exoskeleton

The control system and the power system are mounted at the waist mechanism. Users were not burdened with a backpack if they present impaired sensation over their trunk. Furthermore, the powered exoskeleton was designed for multiple users in a hospital or rehabilitation center. As shown in Fig. 4, the size of the thigh, shank, and waist is adjustable. The powered exoskeleton has 2 torso braces to fix the patients’ torso in the waist open/close mechanism by using the chest belt and waist belt. The torso braces are also used to help patients maintain an upright torso. Each leg has 3 braces, with 2 braces at the thigh and 1 brace at the shank. These braces support the patients’ weight and carry the patients’ legs during walking. The locations of the braces are also adjustable to suit different body types. In addition, the powered exoskeleton is equipped with a pair of sandal-type shoes; thus, patients can wear their own shoes and simply place them into the robot shoes. The sandal-type shoes are half-type shoes that facilitate the motion of the foot off the ground more easily. Also, the sandal-type shoe connects to the exoskeleton robot with a flexible support and a drivable and frictional ankle joint. The flexible support can be bent to keep the contact of the sandal-type shoe and ground when the user shifts his/her center of mass laterally. The drivable and fictional ankle joint can also fit to the inclination of ground and prevent the foot-drag. The detailed design of the powered exoskeleton is described in a patent [18].

### Clinical study

#### Participants

The inclusion criteria were as follows: 1) individuals with a thoracic or lumbar complete SCI, 2) the range of motion of the upper extremities should be within the normal range, 3) the strength of the upper extremities should be at least 5 (graded according to a manual muscle test), 4) ability to maintain the sitting position with hand support for ≥30 s without losing balance, and 5) ability to walk using a KAFO with a walker or forearm crutches. The exclusion criteria were as follows: 1) spasticity of the lower extremities > 3 (evaluated according to the Modified Ashworth Scale) [19], 2) with a previous osteoporosis diagnosis, and 3) any recent musculoskeletal injury or fractures, open wounds, or contractures/deformities of the lower extremities that would affect wearing and walking with the powered exoskeleton.

#### Training protocol

An 8-week of gait training using the powered exoskeleton (1 h/session, 2 sessions/week) was provided to equip the participants with the skills required to perform sit-to-stand and walking. The training protocol is described in a previous study and is also illustrated in the Additional file 1 [20].

The participants started the training by practicing sit-to-stand movement. The emphasis was placed on learning proper trunk leaning and appropriate timing for crutch-pushing, which refers to the pushing of the crutches to facilitate momentum transfer during the seat-off phase of sit-to-stand [21]. Before starting to walk, the participants had to first learn proper weight-shifting skills in anteroposterior and mediolateral directions while standing with the assistance of forearm crutches. Such training is essential to executing proper weigh-shifting to ensure continuous steps during walking. The individuals proceeded to practice, taking 1 or 2 steps at a time once they had learned weight-shifting skills.

A physical therapist provided verbal instruction to the participants throughout the practice sessions. Feedback from a postural mirror and video recordings were provided as necessary. To rapidly adapt to the powered exoskeleton, the participants practiced sit-to-stand and walking with parallel bars for additional assistance and stability before proceeding to the forearm crutches. By the end of 8-week training program, both participants could walk 6 steps with “supervision” by the physical therapist. By the time of the clinical evaluation, they could perform both sit-to-stand and walking with the powered exoskeleton with minimal assistance from the physical therapist.

#### Clinical evaluation

All clinical evaluation were administered before the training and after the 8 weeks of gait training program using the powered exoskeleton.

To ensure the participants did not have osteoporosis which might affect the gait training [22], bone mineral density of the lumbar vertebrae and proximal femur was measured using dual-energy X-ray absorptiometry (Lunar iDXA, General Electric Company, USA). The timed up-and-go test [23], 10-m walk test [24], and 6-min walk test [25] were performed to evaluate the mobility performance of using the powered exoskeleton.

The timed up-and-go test has been shown to be a reliable and valid test for quantifying functional mobility in elderly people [23]. The timed up-and-go test is a clinical test for evaluating a person’s mobility [23]. Specifically, it evaluates an individual’s ability to stand, walk, turn, and sit. The completion of a series of such movements in a timely manner requires balance control. During the test, the participants were required to stand up, walk 3 m, turn, walk back, and sit down as quickly as possible. The time for completion of the test was recorded.

During the 10-m walk test, the participants walked for 10 m at a comfortable speed, and the time for completion of the test was recorded. They walked 2–3 steps before and after passing the start and end of the 10-m mark.

The 6-min walk test was used to evaluate the physical capacity and endurance of the participants when walking using a KAFO and the powered exoskeleton. During this test, the participants were required to continuously walk at their preferred speed with a KAFO and as quickly as they could manage with the powered exoskeleton for as far as possible in 6 min. A physical therapist walked behind the participant the whole time to supervise or provide assistance as necessary. The distance and number of steps completed by the participant over the 6 min were recorded.

All the tests were performed twice, once with a KAFO and once with the powered exoskeleton. In addition, to evaluate the device feasibility and safety, the following measurements were taken: time taken to don and doff the device, level of assistance required [26], and the incidence of falls or loss of balance.

To prevent fatigue, evaluations were conducted on 2 consecutive days. Vital signs with the participants in a resting state were measured to ensure that the participants were in the same health condition while performing the tests. Because both of the participants used a walker to assist them with walking while wearing a KAFO on a daily basis, forearm crutches were not mandated while performing the timed up-and-go test, 10-m walk test, and 6-min walk test. Therefore, both of the participants used a walker for assistance while wearing a KAFO to perform the 3 tests. Their blood pressure and heart rate were measured before and after each test to ensure that the participants were in a stable condition before proceeding to the next test. To evaluate the level of exertion perceived by the participants using a KAFO and the powered exoskeleton, the rated perceived exertion (RPE) was obtained upon completion of the 6-min walk test.

## Results

Two participants were included in this pilot study. The basic data before training was listed in Table 1. Both participants were 2 men aged 38–40 years who had complete spinal cord injury between T4 and T10 (Grade A according to the American Spinal Injury Association Impairment Scale). This two participants had sufficient experience with walking regularly with a KAFO as their routine exercise for 30–60 min/session and at least 5 sessions/wk, for approximately 1–2 years before participating in this study. They continued their routine walking exercise with a KAFO throughout the powered exoskeleton gait training.

**Table 1:** Basic data of the participants

	Participant 1	Participant 2
Age (years)	38	36
Sex	Male	Male
Height (cm)	180.0	170.0
Weigh (kg)	58.0	62.3
ASIA scale^a^	T10, A	T4, A
Anthropometric measures (cm)		
Trunk length	32.0	35.0
Trunk depth	16.3	18.2
Thigh length (left/right)	42.0/42.5	40.0/40.0
Thigh circumference (left/right)	37.5/38.3	38.2/37.4
Shank length (left/right)	47.3/48.0	45.0/45.5
Shank circumference (left/right)	25.7/24.2	28.0/27.6
Manual Muscle Testing		
Shoulder extension (left/right)	5/5	5/5
Shoulder depression (left/right)	5/5	5/5
Elbow extension (left/right)	5/5	5/5
Elbow flexion (left/right)	5/5	5/5
Sitting balance^b^	4	2

^a^*ASIA* American Spinal Injury Association Impairment Scale

^b^Part of Motor Assessment Scale (Part III, the full score is 6, the higher score, the better sitting balance)

### Feasibility and safety

With the open/closed design of the powered exoskeleton, users can independently transfer themselves in and out of the device. Both of the participants could don and doff the powered exoskeleton without any assistance. Both participants could don and doff the powered exoskeleton within 5 min, which was faster than using a KAFO (Table 2). The results showed that the users could use the powered exoskeleton independently and don and doff the device in a timely manner, indicating that the device does not impose an additional time burden on the user. A main reason for giving up the use of long leg calipers is that putting them on is time consuming [27]. Thus, it is imperative that ease of use be considered when designing the assistive device.

**Table 2:** Functional performance when using the powered exoskeleton with forearm crutches and a KAFO with a walker

	Participant 1		Participant 2	
Assistive Device	EXO	KAFO	EXO	KAFO
Time to don and doff (sec)	220	250	250	260
Level of assistance during sit-to-stand^a^	5	6	5	6
Timed up-and-go test				
Time needed (sec)	70	182	110	128
Level of assistance^a^	4	4	4	6
10-m walk test				
Time needed (sec)	52	155	76	126
Steps (numbers)	25	52	35	37
Speed (m/s)	0.19	0.06	0.13	0.07
Level of assistance^a^	4	5	4	4
6-min walk test				
Distance (m)	69.33	19.8	55.25	23.39
Steps (numbers)	169	112	183	96
Speed (m/s)	0.193	0.055	0.15	0.065
RPE^b^	6	8	5	8

*EXO* Powered Lower Limb Exoskeleton Robot, *KAFO* knee–ankle–foot orthosis

^a^Level of Assistance:

7: Complete independence;

6: Modified independence (requiring the use of a device but no physical help);

5: Supervision (requiring only standby assistance or verbal prompting or help with set-up);

4: Minimal assistance (requiring incidental hands-on help only; participants perform > 75% of the task);

3: Moderate assistance (participants still perform 50%–75% of the task);

2: Maximal assistance (participants provide less than half of the effort [25%–49%]);

1: Total assistance (participants contribute < 25% of the effort or are unable to perform the task)

^b^RPE: Rated Perceived Exertion scale from 0 (*nothing at all*) to 10 (*very, very heavy*)

### Mobility and balance function

Both participants successfully completed sit-to-stand movements without any falls and level of assistance was lower when using the powered exoskeleton when compared to using a KAFO. Table 2 also presents the results of the timed up-and-go test, 10-m walk test, and 6-min walk test. In general, both participants completed all 3 functional tests faster when wearing the powered exoskeleton than when wearing a KAFO (i.e., approximately 1.2–3.5 times faster).

As shown in Table 2, the participants completed the timed up-and-go test 2-fold faster when using the powered exoskeleton than when using a KAFO. The results suggest that the powered exoskeleton provides functional mobility to the user. The 10-m walk test evaluates an individual’s walking ability. The participants are required to walk in a straight line as quickly as possible. The test results thus reflect an individual’s steady walking performance. Overall, the participants in the present study walked 2–3-fold faster when using the powered exoskeleton than when using a KAFO (Table 2).

### Endurance and perceived exertion

The participants’ endurance while wearing the powered exoskeleton was evaluated through the 6-min walk test. Both participants successfully completed the test without any discomfort. During the test, the distance covered and walking speed were 2–3-fold longer and faster, respectively, when using the powered exoskeleton than when using a KAFO (Table 2). Both participants perceived lower exertion during the 6-min walk test while using the powered exoskeleton (RPE: 5–6) than while using a KAFO (RPE: 8).

## Discussion

We evaluated the feasibility and safety of the powered lower limb exoskeleton robot in this study. The results showed that our newly developed powered lower limb exoskeleton robot prototype assisted the individuals with SCI accomplishing functional activities such as sit-to-stand and walking safely and independently. The users did not report any discomfort or adverse effects such as skin abrasions. The users provided positive feedback after using the device, including greater ease with donning and doffing and lower perceived exertion when using the powered exoskeleton than when using a KAFO.

The major concern for users is the safety of the exoskeleton robot. Overall, a zero fall incidence was reported while walking with both the powered exoskeleton and a KAFO. In addition, the users reported no adverse effects, such as skin abrasions, during training or testing. A notable feature of the design of the powered exoskeleton is the remote control embedded underneath the crutches, which promotes user independence. The participants could easily initiate and terminate movements such as sit-to-stand and walking by using the remote control. These findings suggest that the powered exoskeleton is a safe and practical device that can assist people with SCI in completing activities of daily living.

For individuals with SCI, bone mineral density loss has been reported as a complication because of the lack of stimulation from weight-bearing activities after injury onset [28, 29]. In the current study, we monitored the bone mineral density of the participants before and after training. The bone mineral densities demonstrated an increasing trend implying a potential training benefit of the powered exoskeleton for people with SCI. Long-term follow-up of the progression of changes in bone density is required in order to determine the training effect of the powered exoskeleton on bone health.

The timed up-and-go test is a clinical functional test for evaluating a person’s mobility. [23] Our study shows that the individuals with a complete SCI completed the timed up-and-go test 2-fold faster when using the powered exoskeleton than when using a KAFO. The results suggest that the powered exoskeleton provides functional mobility to the user. Additional studies should evaluate whether users have greater dynamic balance control when using the powered exoskeleton than when using a KAFO by investigating the smoothness of the walking pattern during the timed up-and-go test. These findings suggest that compared with current standard assistive walking devices, such as KAFOs, the powered exoskeleton can assist people with complete SCI to achieve efficient functional walking without imposing additional energy demands.

The challenge of control the initiation and termination of any powered exoskeleton movement is that the individuals with SCI needs to press the controller bottoms and still needs their hands to hold on to the forearm crutches to maintain their upright balance. This is a multitasking condition and could be difficult for the individuals with poor upright balance [30]. Our design of controller bottoms can resolve this dilemma because the remote controllers were embedded in the handle part of the forearm crutches for each hand. The individuals with SCI did not need to free one hand to press the controller bottoms on a watch or a wrist band. Therefore, they could easily start/stop the powered exoskeleton without looking at the bottoms, nor stop walking so that their upright balance was not compromised during the manipulations of the controller bottoms.

In summary, we provided a standard training procedure for using the powered exoskeleton to help participants acquire the skills required for performing functional activities such as sit-to-stand and walking. This is the first study to establish a standard training protocol using the powered lower limb exoskeleton robot. Future studies should recruit more participants to evaluate the practicability of the training protocol.

## Conclusions

The safety, feasibility, and mobility of our newly developed powered lower limb exoskeleton robot were evaluated in this study. The findings demonstrate that individuals with complete SCI can independently don and doff the powered exoskeleton and can perform sit-to-stand and walking faster and with lower exertion than they can when using a KAFO. No adverse effects, such as skin abrasions, or discomfort, were reported by the participants while using the powered exoskeleton. These findings suggest that the powered exoskeleton is safe, easy to use, and can assist individuals with SCI in efficiently accomplishing daily functional activities. Moreover, during an interview at the end of training, one participant mentioned that he felt that it gradually became easier for him to pass stool manually after constantly walking using the powered exoskeleton, which is an interesting finding that should be further investigated.

## Additional file

Additional file 1: Exoskeleton training manual for individuals with spinal cord injury. (DOCX 4244 kb)

## Abbreviations

EMG: Electromyography
KAFO: Knee–ankle–foot orthosis
RPE: Rated perceived exertion
SCI: Spinal cord injury

## References

[1] Waters RL, Lunsford BR (1985). Energy cost of paraplegic locomotion. J Bone Joint Surg Am.

[2] Fatone S (2006). A review of the literature pertaining to KAFOs and HKAFOs for ambulation. J Prosthet Orthot.

[3] Mooney LM, Herr HM (2016). Biomechanical walking mechanisms underlying the metabolic reduction caused by an autonomous exoskeleton. J NeuroEng Rehabil.

[4] Young A, Foss J, Gannon H, Ferris DP. Influence of power delivery timing on the energetics and biomechanics of humans wearing a hip exoskeleton. Front Bioeng Biotechnol. 2017;5. https://www.ncbi.nlm.nih.gov/pmc/articles/PMC5340778/. 10.3389/fbioe.2017.00004 PMC5340778 28337434

[5] Requejo PS, Wahl DP, Bontrager EL, Newsam CJ, Gronley JK, Mulroy SJ, Perry J (2005). Upper extremity kinetics during Lofstrand crutch-assisted gait. Med Eng Phys.

[6] Sie IH, Waters RL, Adkins RH, Gellman H (1992). Upper extremity pain in the postrehabilitation spinal cord injured patient. Arch Phys Med Rehabil.

[7] Wang TY, Pao JL, Yang RS, Jang JS, Hsu WL (2015). The adaptive changes in muscle coordination following lumbar spinal fusion. Hum Mov Sci.

[8] Yang WC, Cheng CH, Wang HK, Lin KH, Hsu WL (2015). Multi-muscle coordination during a challenging stance. Eur J Appl Physiol.

[9] Ferrati F, Bortoletto R, Menegatti E, Pagello E (2013). Socio-economic impact of medical lower-limb exoskeletons. 2013 IEEE workshop on advanced robotics and its social impacts; 7–9 Nov. 2013.

[10] Banala SK, Agrawal SK, Fattah A, Krishnamoorthy V, Hsu WL, Scholz J, Rudolph K (2006). Gravity-balancing leg orthosis and its performance evaluation. IEEE Trans Robot.

[11] Agrawal SK, Banala SK, Fattah A, Sangwan V, Krishnamoorthy V, Scholz JP, Hsu WL (2007). Assessment of motion of a swing leg and gait rehabilitation with a gravity balancing exoskeleton. IEEE Trans neural Syst Rehabil Eng.

[12] Tsukahara A, Kawanishi R, Hasegawa Y, Sankai Y (2010). Sit-to-stand and stand-to-sit transfer support for complete paraplegic patients with robot suit hal. Adv Robotics.

[13] Esquenazi A, Talaty M, Packel A, Saulino M (2012). The ReWalk powered exoskeleton to restore ambulatory function to individuals with thoracic-level motor-complete spinal cord injury. Am J Phys Med Rehab.

[14] Strausser KA, Kazerooni H (2011). The development and testing of a human machine interface for a mobile medical exoskeleton. 2011 IEEE/RSJ international conference on intelligent robots and systems; 25–30 sept. 2011.

[15] Chang SR, Nandor MJ, Li L, Kobetic R, Foglyano KM, Schnellenberger JR, Audu ML, Pinault G, Quinn RD, Triolo RJ (2017). A muscle-driven approach to restore stepping with an exoskeleton for individuals with paraplegia. J NeuroEng Rehabil.

[16] Kim SM, Lee SY, Kang HC, Jeong JH (2009). Study of knee and hip joints’ moment estimation by biomechanical simulation during various motion changes. Lecture Notes in Engineering and Computer Science.

[17] Crowell HP, Boynton AC, Mungiole M (2002). Exoskeleton power and torque requirements based on human biomechanics. Army research lab aberdeen proving ground md.

[18] Teng MC, Tsai YJ: Walking assist device. In Book walking assist device (Editor ed.^eds.). City; 2015.

[19] Bohannon RW, Smith MB (1987). Interrater reliability of a modified Ashworth scale of muscle spasticity. Phys Ther.

[20] Yang W, Hsu W, Wu C, Hu J, Mao H, Tang P (2013). A short-term gait training protocol for using the powered exoskeleton in people with spinal cord injury. Formos J Phys Ther.

[21] Schenkman M, Berger RA, Riley PO, Mann RW, Hodge WA (1990). Whole-body movements during rising to standing from sitting. Phys Ther.

[22] Hsu WL, Chen CY, Tsauo JY, Yang RS (2014). Balance control in elderly people with osteoporosis. J Formos Med Assoc.

[23] Podsiadlo D, Richardson S (1991). The timed “up & go”: a test of basic functional mobility for frail elderly persons. J Am Geriatr Soc.

[24] van Hedel HJ, Wirz M, Dietz V (2005). Assessing walking ability in subjects with spinal cord injury: validity and reliability of 3 walking tests. Arch Phys Med Rehabil.

[25] Enright PL (2003). The six-minute walk test. Respir Care.

[26] Davidoff GN, Roth EJ, Haughton JS, Ardner MS (1990). Cognitive dysfunction in spinal cord injury patients: sensitivity of the functional independence measure subscales vs neuropsychologic assessment. Arch Phys Med Rehabil.

[27] Hawran S, BieringSorensen F (1996). The use of long leg calipers for paraplegic patients: a follow-up study of patients discharged 1973-82. Spinal Cord.

[28] Bieringsorensen F, Bohr HH, Schaadt OP (1990). Longitudinal-study of bone-mineral content in the lumbar spine, the forearm and the lower-extremities after spinal-cord injury. Eur J Clin Investig.

[29] Clasey JL, Janowiak AL, Gater DR (2004). Relationship between regional bone density measurements and the time since injury in adults with spinal cord injuries. Arch Phys Med Rehab.

[30] Hsu WL, Scholz JP (2012). Motor abundance supports multitasking while standing. Hum Mov Sci.

