# Supplementary material for: The effects of gait training using powered lower limb exoskeleton robot on individuals with complete spinal cord injury
Source: J Neuroeng Rehabil. 2018 Mar 5;15:14. doi: 10.1186/s12984-018-0355-1 (PMC5838988; doi:10.1186/s12984-018-0355-1)
Supplement: Supplementary file 1 — Exoskeleton training manual for individuals with spinal cord injury. (DOCX 4244 kb) [file 12984_2018_355_MOESM1_ESM.docx]

# Exoskeleton Training Manual for Individuals with Spinal Cord Injury

## Table of Contents

[**Indications** **2**](#_Toc401834914)

[**Safety Considerations and Precautions** **2**](#_Toc401834915)

[**Contraindications** **3**](#_Toc401834916)

[**Settings and Equipment** **4**](#_Toc401834917)

[**Training Protocol** **5**](#_Toc401834918)

[The First Session 6](#_Toc401834919)

[The Second Session 8](#_Toc401834920)

[The Third Session 11](#_Toc401834921)

[The Fourth Session 13](#_Toc401834922)

[The Fifth Session 14](#_Toc401834923)

[The Sixth Session 16](#_Toc401834924)

[The Seventh Session 18](#_Toc401834925)

[The Eighth Session 20](#_Toc401834926)

[**Evaluation** **21**](#_Toc401834927)

[**Demonstration of the Functional Movements** **24**](#_Toc401834928)

[Practice Sit-to-Stand and Stand-to-Sit with parallel bars 24](#_Toc401834929)

[Practice Sit-to-Stand and Stand-to-Sit with forearm crutches 26](#_Toc401834930)

[Practice taking two steps with parallel bars 28](#_Toc401834931)

[Practice walking with forearm crutches 31](#_Toc401834932)

[Three-point Gait vs. Four-point Gait 33](#_Toc401834933)

## Indications

1. Individual with spinal cord injury (SCI) in stable condition for rehabilitation, with intact upper extremity function and strength (manual muscle testing score ≥ 5).
2. With a stable spine (e.g., unstable spine has been fixed via surgical procedures).
3. Ability to maintain sitting balance with arm support ≥ 5 minutes.
4. Familiar with, or a regular user of forearm crutches and/or walker, and can currently walk with forearm crutches or walker for at least 30 minutes.
5. Shoulder range of motion (ROM) within normal limits.

## Safety Considerations and Precautions

1. The therapist should frequently check the user’s skin conditions of the abdomen, lower back, thigh, and anterior tibia areas for redness or abrasions, while the user is using the ITRI-EXO.
2. Avoid directly applying the strap over the wound and/or insertion of the drainage tube.
3. Be cautious with postural hypotension.
4. Be cautious with osteoporosis or heterotopic ossification.
5. Pay attention to the user’s sitting balance (the user must be able to maintain sitting balance with arm support).
6. Belly fat could affect the fixation of the trunk straps.
7. Abnormally increased muscle tone (spasticity) in the lower extremities could affect the movement of the robotic legs. The user with spasticity in his/her lower extremities should not use the exoskeleton for training to avoid injury.

## Contraindications

1. Impaired cognitive function.
2. Leg length discrepancy, lower extremity contracture, or limited ROM of the lower extremities.
3. Impaired upper extremity function (e.g., strength or ROM).
4. Abnormally increased muscle tone (spasticity) in the lower extremities that prevents the user from standing.

## Settings and Equipment

The following items/equipment are needed for the training:

1. Parallel bars
2. Postural mirror or projection device. To provide the user with visual feedback during the training.
3. Movable stool (lockable) / or wheel chair (with removable armrests)

## Training Protocol

The example shown below is an 8-session training protocol to allow the user acquire skills required to perform **sit-to-stand** and **straight-line walking.** The training protocol is designed for the user who meets the following conditions:

1. Ability to maintain sitting balance with arm support.
2. Familiar with, or a regular user of forearm crutches and/or walker, and has experience walking with those assistive devices.

(**Note:** Additional training sessions will be required for the user who does not meet the abovementioned conditions.)

**Table 1: Sample eight-session training protocol**

| **Training session** | **1** | **2** | **3** | **4** | **5** | **6** | **7** | **8** |
| --- | --- | --- | --- | --- | --- | --- | --- | --- |
| Evaluation |  |  |  |  |  |  |  |  |
| Sit-to-Stand and Stand-to-Sit with parallel bars |  |  |  |  |  |  |  |  |
| Sit-to-Stand and Stand-to-Sit with forearm crutches |  |  |  |  |  |  |  |  |
| Walking with parallel bars |  |  |  |  |  |  |  |  |
| Walking with forearm crutches |  |  |  |  |  |  |  |  |
| Turning |  |  |  |  |  |  |  |  |

(**Note:** Each training session lasts for 50-60 minutes, including warm-up and two breaks (5 min each). Warm-up exercise is necessary to prevent muscular injury (e.g., shoulder impingement syndrome, carpel tunnel syndrome…etc).

### The First Session

**Goals:**

1. Take body measurements. Evaluate donning and doffing of the ITRI-EXO.
2. Evaluate the user’s condition to determine if he/she meets the criteria for ITRI-EXO training.
3. Provide the user with the information about how the ITRI-EXO works.

**Sample training plan:**

| **Type of Activity** | **Duration** | **Content** |
| --- | --- | --- |
| Evaluation | 15 | Refer to “Evaluation” session (page 21) |
| Break | 5 |  |
| Device fitting | 15 | 1. In sitting position, make sure the hip and knee joints of the ITRI-EXO are aligned well with those of the user’s. 2. Check the alignment again in standing position. |
| Practice Sit-to-Stand and Stand-to-Sit with parallel bars | 5 | 1. Provide the user with a video demonstration before the training. 2. Key points for instruction: 3. Keep the trunk upright during the movement. 4. Memorize the motion and speed of the robotic legs. |
| Break | 5 |  |
| Practice Sit-to-Stand and Stand-to-Sit with parallel bars | 5 |  |
| Practice two steps with parallel bars | 10 | 1. Provide the user with a video demonstration before the training. 2. Key points for instruction: 3. Keep the trunk upright when the swing leg (i.e., the leg which is about to move forward will be called swing leg) is moving forward. 4. Shift your body weight to the stance leg when the swing leg is moving forward. 5. When the swing leg is completely straight and touches the ground, move both hands forward along the bars, and shift your body weight forward. 6. Use the mirror or video recordings to provide user the visual feedback. |

### The Second Session

**Goals:**

1. Start practicing Sit-to-Stand and Stand-to-Sit with forearm crutches.
2. Start practicing stepping.

**Sample training plan:**

| **Type of Activity** | **Duration** | **Content** |
| --- | --- | --- |
| Transfer into and don the device | 5 |  |
| Practice Sit-to-Stand and Stand-to-Sit with parallel bars | 5 | 1. Key points for instruction: 2. Keep the trunk upright during the movement. 3. Memorize the motion and speed of the robotic legs. |
| Practice Sit-to-Stand and Stand-to-Sit with forearm crutches | 5 | 1. Provide the user with a video demonstration before the training. 2. Key points for instruction: 3. Keep the trunk upright during the movement. 4. Do not exert too much force on the crutches. Use the crutches to keep your balance during the movement. 5. Before practicing sit-to-stand movement, the user can familiar him/herself with the following movement: Maintain standing balance while moving the crutches forward and backward (see figure below).   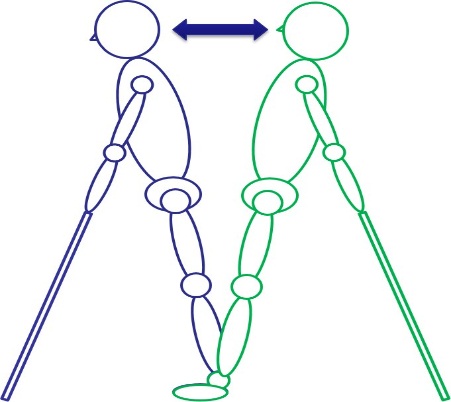 |
| Break | 5 |  |
| Practice Sit-to-Stand and Stand-to-Sit with forearm crutches | 10 |  |
| Practice two steps with parallel bars | 5 | 1. Key points for instruction: 2. Keep the trunk upright when the swing leg is moving forward. 3. Shift your body weight to the stance leg when the swing leg is moving forward. 4. When the swing leg is completely straight and touches the ground, move both hands forward along the bars, and shift your body weight forward. |
| Break | 5 |  |
| Practice two steps with parallel bars | 15 |  |
| Doff and transfer out of the device | 5 |  |

### The Third Session

**Goals:**

1. Familiarize yourself with the skills required to perform Sit-to-Stand and Stand-to-Sit with forearm crutches.
2. Familiarize yourself with the skills required to perform stepping movement.

**Sample training plan:**

| **Type of Activity** | **Duration** | **Content** |
| --- | --- | --- |
| Transfer into and don the device | 5 |  |
| Practice sit-to-stand and stand-to-sit with forearm crutches | 10 | 1. Key points for instruction: 2. Keep the trunk upright during the movement. 3. Do not exert too much force on the crutches. Use the crutches to keep your balance during the movement. |
| Break | 5 |  |
| Practice two steps with parallel bars | 10 | 1. Key points for instruction: 2. Keep the trunk upright when the swing leg is moving forward. 3. Shift your body weight to the stance leg when the swing leg is moving forward. 4. When the swing leg is completely straight and touches the ground, move both hands forward along the bars, and shift your body weight forward. |
| Practice a few steps with parallel bars | 5 | 1. Practice more steps once the user can manage to take two steps successfully. |
| Break | 5 |  |
| Practice a few steps with parallel bars | 15 |  |
| Doff and transfer out of the device | 5 |  |

### The Fourth Session

**Goals:**

1. Familiarize yourself with the skills required to perform stepping movement.

**Sample training plan:**

| **Type of Activity** | **Duration** | **Content** |
| --- | --- | --- |
| Transfer into and don the device | 5 |  |
| Practice a few steps with parallel bars | 10 |  |
| Break | 5 |  |
| Practice a few steps with parallel bars | 15 |  |
| Break | 5 |  |
| Practice a few steps with parallel bars | 15 |  |
| Doff and transfer out of the device | 5 |  |

### The Fifth Session

**Goals:**

1. Start practicing stepping movement with forearm crutches.

**Sample training plan:**

| **Type of Activity** | **Duration** | **Content** |
| --- | --- | --- |
| Transfer into and don the device | 5 |  |
| Practice a few steps with parallel bars | 10 |  |
| Break | 5 |  |
| Practice two steps with forearm crutches | 15 | 1. Before practicing, the user can familiar him/herself with the following movements while wearing the ITRI-EXO: Maintain standing balance while moving the crutches forward and backward; Maintain standing balance while shifting your body weight from side to side. 2. Key points for instruction: 3. Keep the trunk upright when the swing leg is moving forward. 4. Shift your body weight to the stance leg when the swing leg is moving forward. 5. Do not exert too much force on the crutches. Use the crutches to keep your balance during the movement. 6. When the swing leg is completely straight and touches the ground, move the crutches forward, and shift your body weight forward. 7. Use the mirror or video recordings to provide visual feedback. |
| Break | 5 |  |
| Practice two steps with forearm crutches | 15 |  |
| Doff and transfer out of the device | 5 |  |

### The Sixth Session

**Goals:**

1. Start practicing stepping movement with forearm crutches.

**Sample training plan:**

| **Type of Activity** | **Duration** | **Content** |
| --- | --- | --- |
| Transfer into and don the device | 5 |  |
| Practice two steps with forearm crutches | 10 | 1. Before practicing, the user can familiar him/herself with the following movement while wearing the ITRI-EXO: Maintain standing balance while moving the crutches forward and backward; Maintain standing balance while shifting your body weight from side to side. 2. Key points for instruction: 3. Keep the trunk upright when the swing leg is moving forward. 4. Shift your body weight to the stance leg when the swing leg is moving forward. 5. Do not exert too much force on the crutches. Use the crutches to keep your balance during the movement. 6. When the swing leg is completely straight and touches the ground, move the crutches forward, and shift your body weight forward. 7. Use the mirror or video recordings to provide visual feedback. |
| Break | 5 |  |
| Practice two steps with forearm crutches | 15 |  |
| Break | 5 |  |
| Practice two steps with forearm crutches | 15 |  |
| Doff and transfer out of the device | 5 |  |

### The Seventh Session

**Goals:**

1. Familiarize yourself with the skills required to perform stepping movement with forearm crutches.

**Sample training plan:**

| **Type of Activity** | **Duration** | **Content** |
| --- | --- | --- |
| Transfer into and don the device | 5 |  |
| Practice two steps with forearm crutches | 10 | 1. Before practicing, the user can familiar him/herself with the following movement while wearing the ITRI-EXO: Maintain standing balance while moving the crutches forward and backward; Maintain standing balance while shifting your body weight from side to side. 2. Key points for instruction: 3. Keep the trunk upright when the swing leg is moving forward. 4. Shift your body weight to the stance leg when the swing leg is moving forward. 5. Do not exert too much force on the crutches. Use the crutches to keep your balance during the movement. 6. When the swing leg is completely straight and touches the ground, move the crutches forward, and shift your body weight forward. 7. Use the mirror or video recordings to provide visual feedback. |
| Break | 5 |  |
| Practice several steps with forearm crutches | 15 | Practice more steps once the user can manage to take two steps successfully. |
| Break | 5 |  |
| Practice several steps with forearm crutches | 10 |  |
| Doff and transfer out of the device | 5 |  |

### The Eighth Session

**Goals:**

1. Familiarize yourself with the skills required to perform stepping movement with forearm crutches.

**Sample training plan:**

| **Type of Activity** | **Duration** | **Content** |
| --- | --- | --- |
| Transfer into and don the device | 5 |  |
| Practice a few steps with forearm crutches | 10 |  |
| Break | 5 |  |
| Practice a few steps with forearm crutches | 10 |  |
| Practice turning with forearm crutches | 5 | Provide the user with a video demonstration before the training. |
| Break | 5 |  |
| Practice turning with forearm crutches | 10 |  |
| Doff and transfer out of the device | 5 |  |

## Evaluation

| **Body Measurements (measurements taken in sitting position)** | | |
| --- | --- | --- |
| 1. Trunk thickness (greater trochanter to the buttocks) (cm) |  | |
|  | **Left** | **Right** |
| 1. Thigh length (greater trochanter to femur lateral epicondyle) (cm) |  |  |
| 1. Thigh circumference (measurement taken 25 cm above the knee joint line) (cm) |  |  |
| 1. Shank length (femur lateral epicondyle to the ground, with shoes on) (cm) |  |  |
| 1. Calf circumference (measurement taken 20 cm below the knee joint line) (cm) |  |  |
| 1. Trunk width (the distance between the 10^th^ ribs) (cm) |  | |
| 1. Height (in lying position) (cm) |  | |
| 1. Weight (kg) |  | |
|  | | |

| **Vital Signs** | **Blood Pressure** | **Heart Rate** |
| --- | --- | --- |
| Resting (Sitting) | ______________ | ______________ |
| Standing | ______________ | ______________ |
|  |  |  |
| **Manual Muscle Testing (Upper Extremities)** | **Left** | **Right** |
| Shoulder extension | ______________ | ______________ |
| Shoulder depression | ______________ | ______________ |
| Elbow extension | ______________ | ______________ |
| Elbow flexion | ______________ | ______________ |
|  |  |  |
| **Shoulder range of motion (ROM)** | **Left** | **Right** |
| Shoulder extension | ______________ | ______________ |
| Shoulder internal rotation | ______________ | ______________ |
| Shoulder external rotation | ______________ | ______________ |

**Sitting Balance Evaluation**

**(*****Motor Assessment Scale, Amended Version 1994, Section “Balance Sitting”)**

|  | - 1. Sits only with support. (Therapist should assist patient into sitting.) |
| --- | --- |
|  | - 1. Sits unsupported for 10 seconds. (Without holding on, knees and feet together, feet can be supported on floor.) |
|  | - 1. Sits unsupported, turns head and trunk to look behind. (Feet supported and together on floor. Do not allow legs to abduct or feet to move. Have hands resting on thighs, do not allow hands to move onto plinth. Turn to each side.) |
|  | - 1. Sits unsupported, turns head and trunk to look behind. (Feet supported and together on floor. Do not allow legs to abduct or feet to move. Have hands resting on thighs, do not allow hands to move onto plinth. Turn to each side.) |
|  | - 1. Sits unsupported, reaches forward to touch floor, and returns to starting position. Feet supported on floor. Do not allow patient to hold on. Do not allow legs and feet to move, support affected arm if necessary. Hand must touch floor at least 10 cm (4 inches) in front of feet. Reach with each arm. |
|  | - 1. Sits on stool unsupported, reaches sideways to touch floor, and returns to starting position. (Feet supported on floor. Do not allow patient to hold on. Do not allow legs and feet to move, support affected arm if necessary. Patient must reach sideways not forward. Reach to both sides.) |

**(Note:** The higher the score, the higher functioning the patient is)

***Reference:** Carr JH, Shepherd RB, Nordholm L, Lynne D. Investigation of a new motor assessment scale for stroke patients. Phys Ther 1985;65:175-80.

## Demonstration of the Functional Movements

### Practice Sit-to-Stand and Stand-to-Sit with parallel bars

| **Sit-to-Stand**   1. Position your chair at one end of the parallel bars. 2. Before initiating the movement, hold on to the parallel bars and keep your trunk upright. 3. As you are being lifted from the chair, hold onto the parallel bars to maintain your balance. 4. Look straight ahead while standing up. | 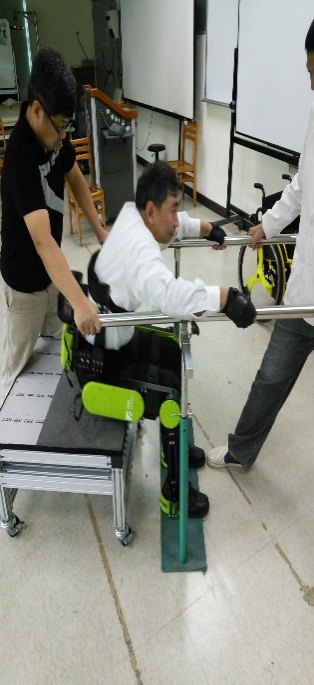 | 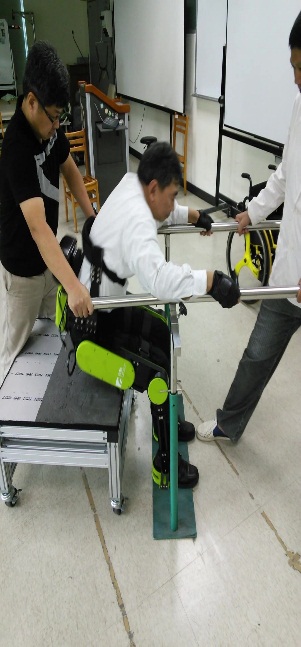 | 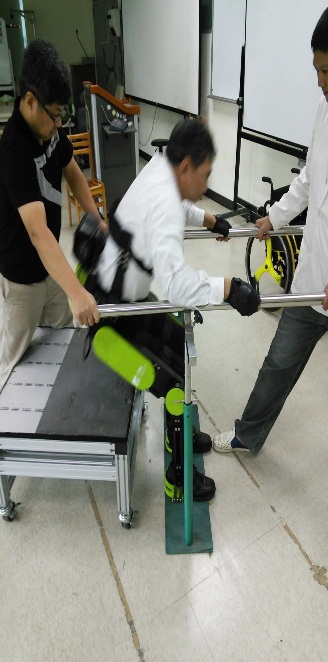 | 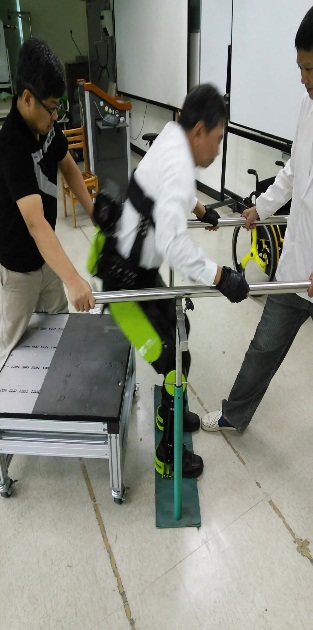 | 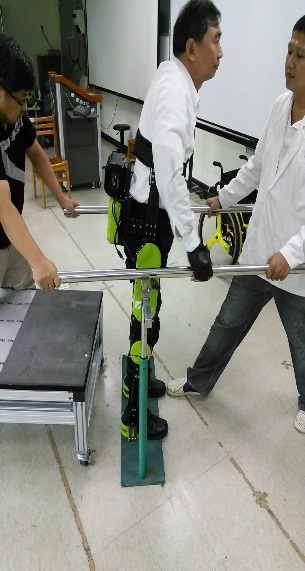 |
| --- | --- | --- | --- | --- | --- |
| **Stand-to-Sit**   1. Place your feet so that the heels are about 10 cm ahead of the chair, to ensure that you will be able to safely land on the chair. 2. Before initiating the movement, hold on to the parallel bars and keep your trunk upright. 3. As you are being lowered onto the chair, hold onto the parallel bars to maintain your balance. 4. Look straight ahead while sitting down. | 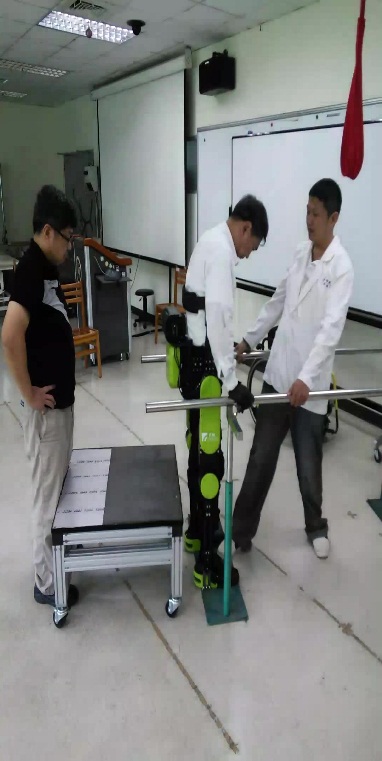 | 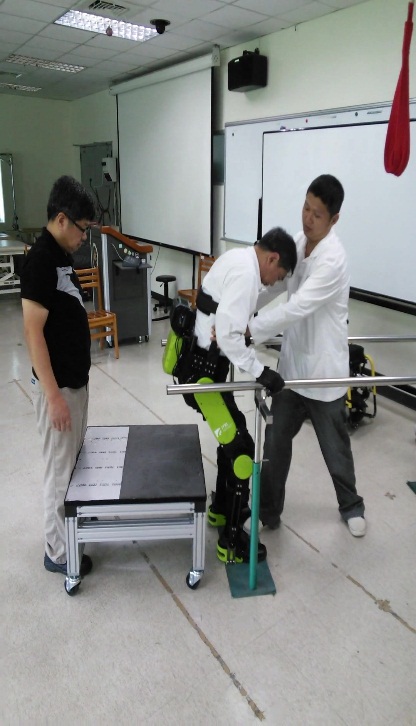 | 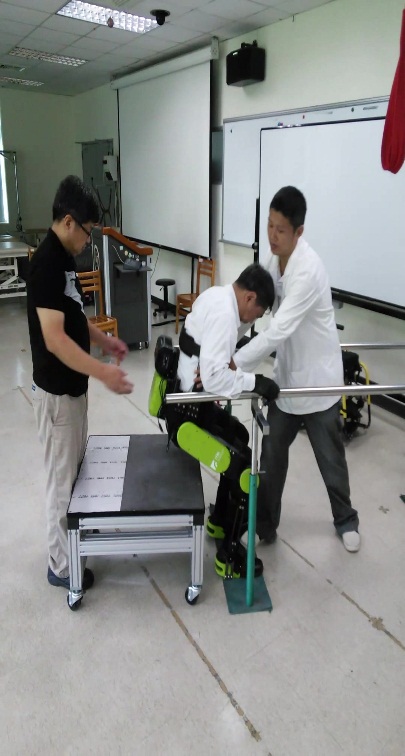 | 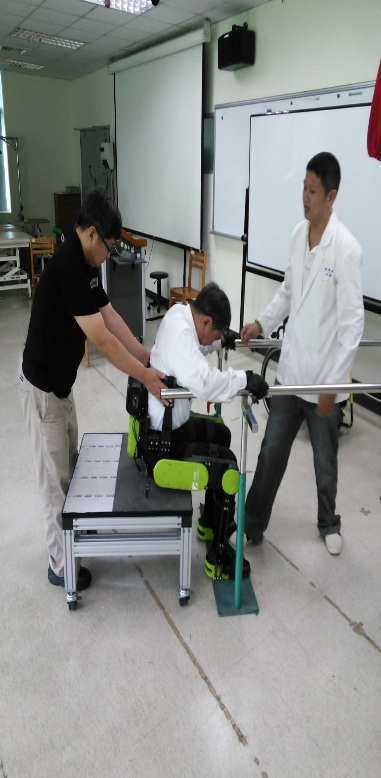 | 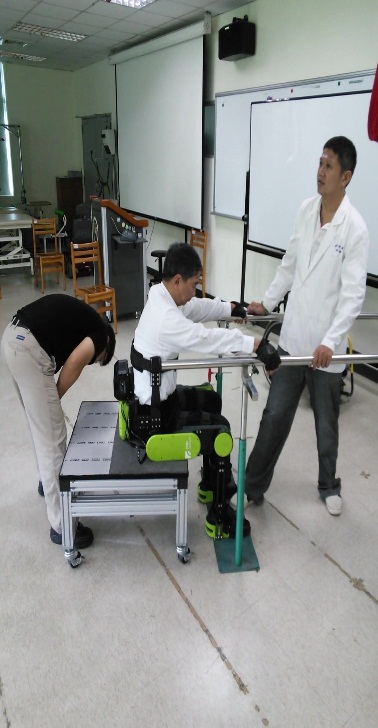 |

### Practice Sit-to-Stand and Stand-to-Sit with forearm crutches

| **Sit-to-Stand**   1. Before initiating the movement, place the crutches 20~30 cm behind the hip joints, and keep your trunk upright. 2. As you are about to be lifted from the chair by the ITRI-EXO, push down on the crutches to maintain your balance and prevent yourself from falling back. Straighten your elbows along the process. 3. As you are being lifted from the chair, push down on the crutches to assist in extending your trunk toward and then past vertical. As you reach a vertical standing position, quickly reposition the crutches anteriorly and assume a balanced standing posture. |  |  |  |
| --- | --- | --- | --- |
| **Stand-to-Sit**   1. Place your feet so that when you pivots on them, you will land on the chair behind. 2. Before initiating the movement, balance on your feet and move the crutches posteriorly. Keep your trunk upright. 3. Keep your trunk upright when initiating the movement. When you are being lowered onto the chair, push down on the crutches to maintain the balance and prevent yourself from falling back. Gradually bend your elbows along the process. |  |  |  |

### Practice taking two steps with parallel bars

| 1. When standing, place your hands on the parallel bars slightly ahead of the hip joint, and keep the your trunk upright. 2. Before initiating the step, push down on the bar on your swing leg side (i.e., the leg which is about to move forward will be called the swing leg; right side as shown in the figure) to shift your body weight to the stance leg (left leg as shown in the figure). 3. Keep your trunk upright when the swing leg moves forward. **Do not shift your body weight forward until the swing leg is completely straight and touches the ground.**   **(Note:** User comfort is the focus of the training. The relative position between hands and hip joints does not have to be overly emphasized during the training.) | 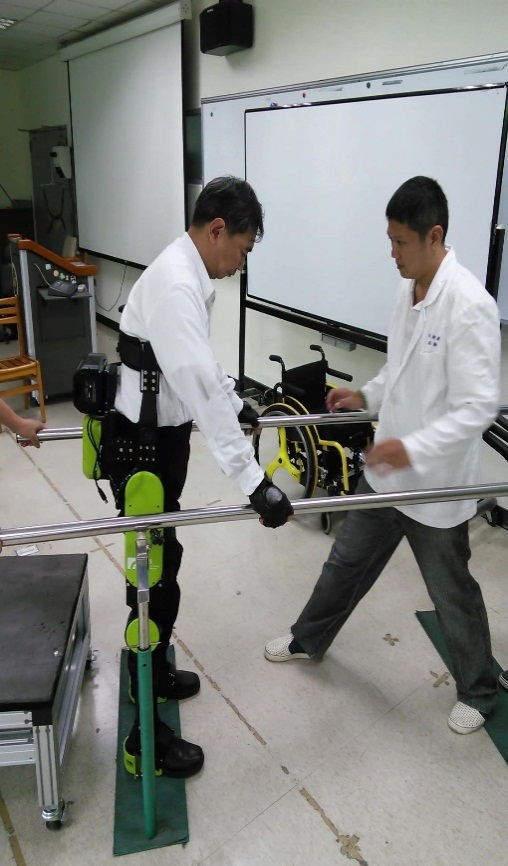 | 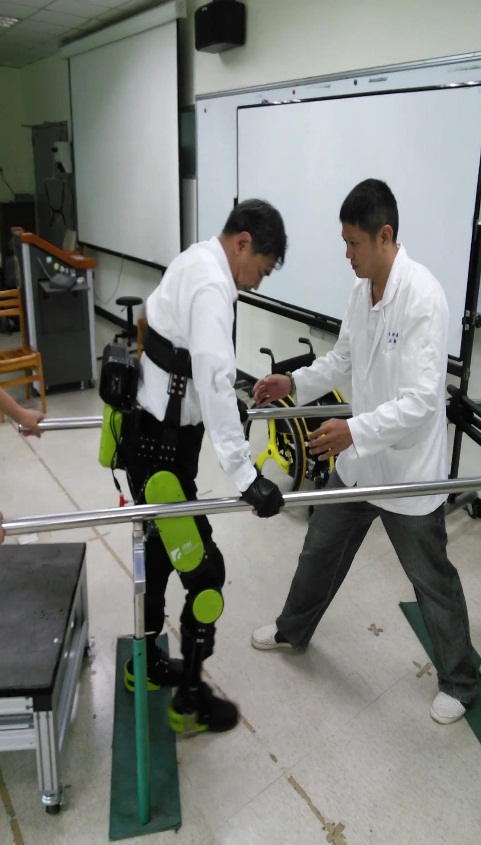 | 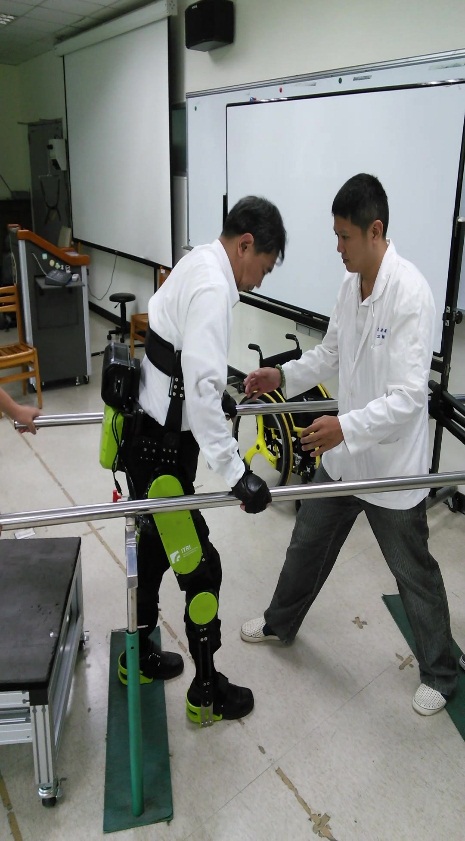 | 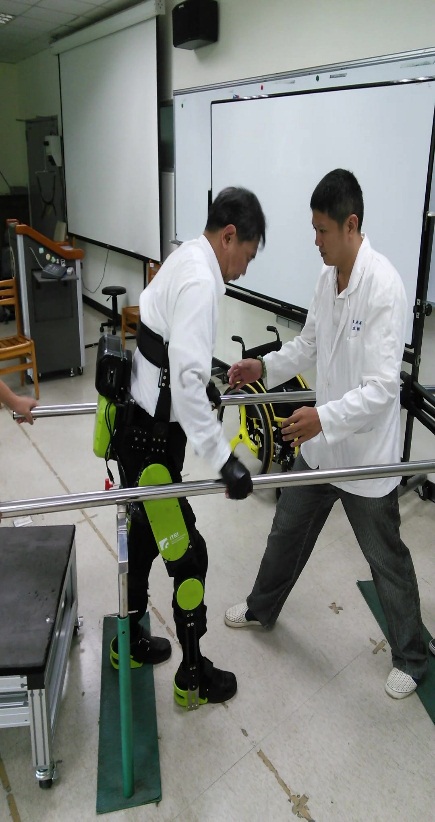 |
| --- | --- | --- | --- | --- |
| 1. After the swing leg touches the ground, move both hands forward along the bars, and shift your body weight from trail limb (left leg as shown in the figure) forward to the lead limb. | 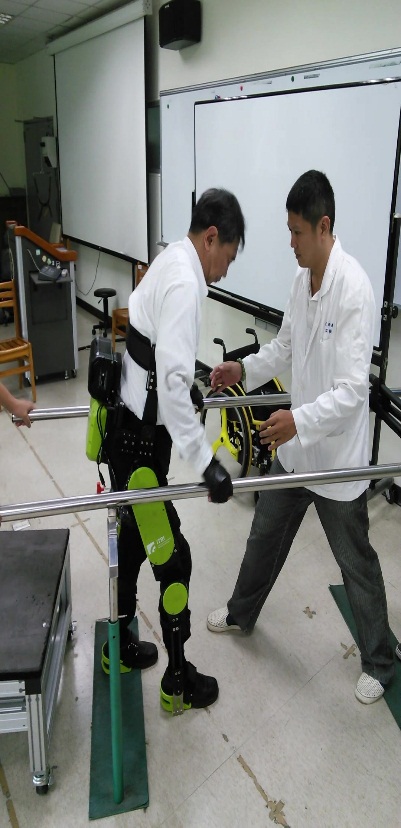 | 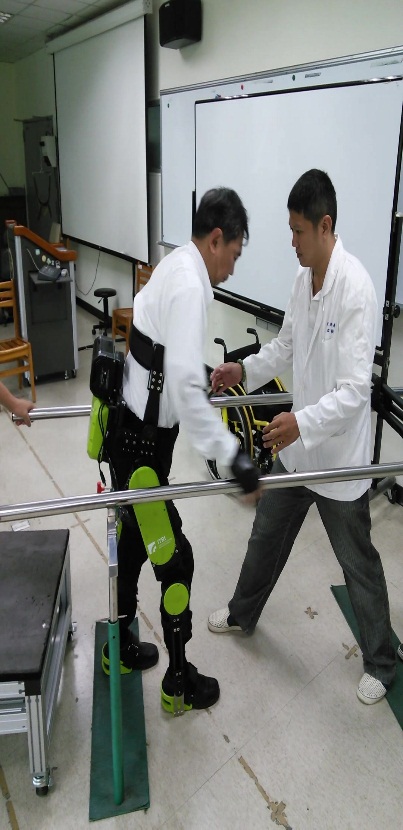 | 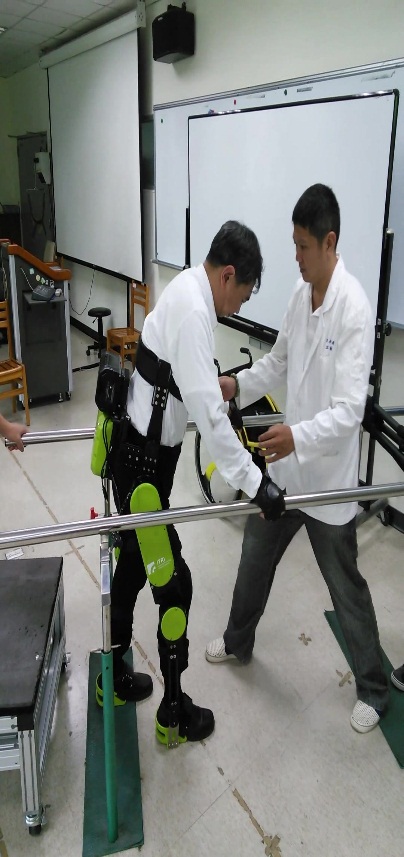 |  |
| 1. Push down on the bar on the stance leg side (left side as shown in the figure) to shift your body weight to the stance leg (right leg as shown in the figure). 2. Keep your trunk upright when the swing leg moves forward. Do not shift your body weight forward until the swing leg is completely straight and touches the ground. 3. After the swing leg touches the ground, move both hands forward along the bars, and shift your body weight from trail limb (right leg as shown in the figure) forward to the lead limb. 4. Repeat steps 4 through 7. | 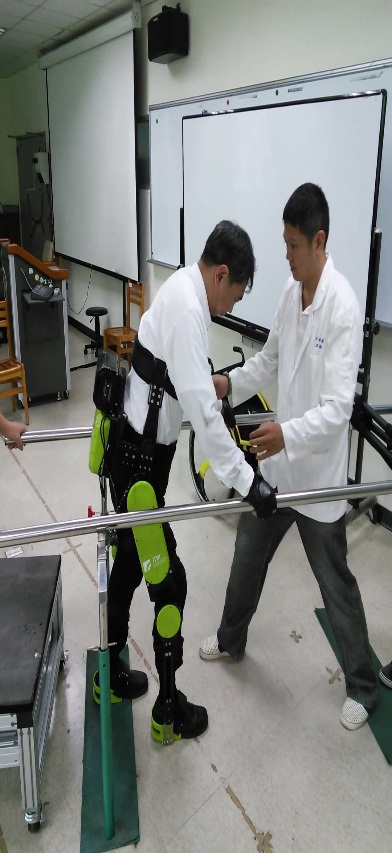 | 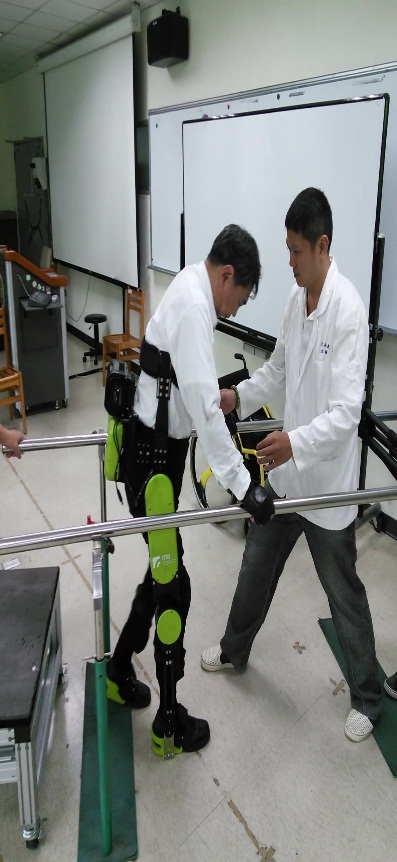 | 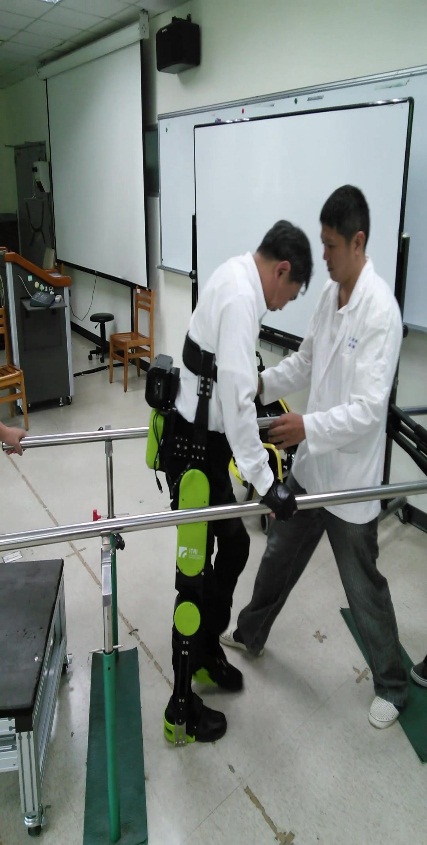 | 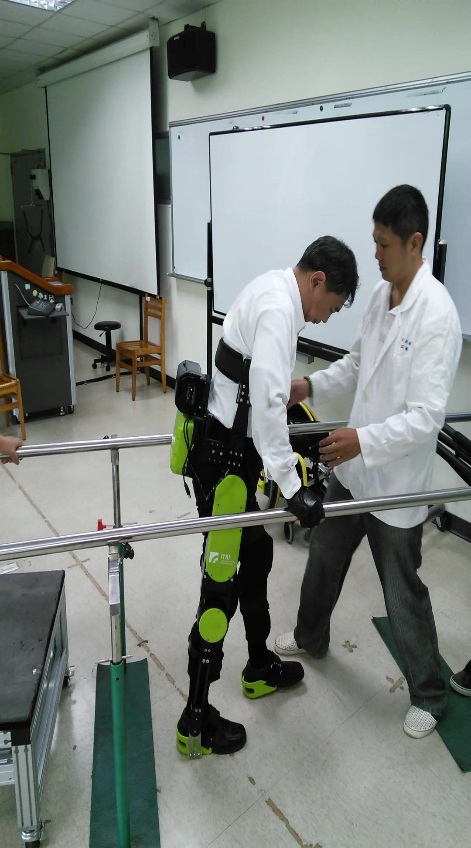 |

### Practice walking with forearm crutches

| 1. Place the crutches ahead and slightly away from your body. Keep your trunk upright. 2. Before initiating the step, push down on the crutch on your swing leg side (right side as shown in the figure) to shift your body weight to the stance leg (left leg as shown in the figure). 3. Keep your trunk upright when the swing leg moves forward. **Do not shift your body weight forward until the swing leg is completely straight and touches the ground.** 4. After the swing leg touches the ground, move the crutches forward ahead of your body, and shift your body weight from trail limb (left leg as shown in the figure) forward to the lead limb.   **(Note:** The black circle in the diagram indicates the crutch position; the gray cross indicates the center of gravity) |      |
| --- | --- |
| 1. Push down on the crutch on the trail limb side (left side as shown in the figure) to shift your body weight to the stance leg (right leg as shown in the figure). 2. Keep your trunk upright when the swing leg moves forward. Do not shift your body weight forward until the swing leg is completely straight and touches the ground. 3. After the swing leg touches the ground, move the crutches forward ahead of your body, and shift your body weight from trail limb (right leg as shown in the figure) forward to the lead limb. 4. Repeat steps 4 through 7. |        |

### Three-point Gait vs. Four-point Gait

| **Crutch Gait Pattern** | **Three-point Gait** | | **Four-point Gait** | |
| --- | --- | --- | --- | --- |
| **Intended Users** | Good trunk stability; Sitting balance score > 3 | | Impaired trunk control, Sitting balance score ≤ 3 | |
|  |  | Keep your body weight even on both feet (keep the center of gravity in the middle between the two feet). |  | Keep your body weight even on both feet (keep the center of gravity in the middle between the two feet). |
|  |  | Before initiating the step, shift your body weight to the stance leg (left leg as shown in the figure). |  | Before initiating the step, shift your body weight to the stance leg (left leg as shown in the figure). |
|  |  | Keep your body weight on the stance leg when the swing leg (right) moves forward. |  | Keep your body weight on the stance leg when the swing leg (right) moves forward. |
|  |  | The swing leg moves forward to the crutch and touches the ground. |  | The swing leg moves forward to the crutch and touches the ground. |
|  |  | Move the crutches forward, and shift your body weight to your lead limb. |  | Move the crutch on your trail limb side forward, and shift your body weight to your lead limb. |
|  |  | Keep your body weight on the stance leg when the swing leg moves forward. |  | Move the crutch on your lead limb side slightly forward to be in line with that on the opposite side.  Keep your body weight on the stance leg when the swing leg moves forward. |
|  |  | The swing leg moves forward to the crutch and touches the ground. |  | The swing leg moves forward to the crutch and touches the ground. |
|  |  | Move the crutches forward, and shift your body weight to your lead limb. |  | Move the crutch on your trail limb side forward, and shift your body weight to your lead limb. |
|  |  | Keep your body weight on the stance leg when the swing leg moves forward. |  | Move the crutch on your lead limb side slightly forward to be in line with that on the opposite side.  Keep your body weight on the stance leg when the swing leg moves forward. |
|  |  | The swing leg moves forward to the crutch and touches the ground. |  | The swing leg moves forward to the crutch and touches the ground. |
